# Longitudinal motor performance development in early adolescence and its relationship to adult success: An 8-year prospective study of highly talented soccer players

**DOI:** 10.1371/journal.pone.0196324

**Published:** 2018-05-03

**Authors:** Daniel Leyhr, Augustin Kelava, Johannes Raabe, Oliver Höner

**Affiliations:** 1 Institute of Sports Science, Eberhard Karls University, Tübingen, Germany; 2 Hector Research Institute of Education Sciences and Psychology, Eberhard Karls University, Tübingen, Germany; 3 Department of Kinesiology, Penn State Altoona, Altoona, Pennsylvania, United States of America; Nanyang Technological University, SINGAPORE

## Abstract

Several talent identification and development (TID) programs in soccer have implemented diagnostics to measure players’ motor performance. Yet, there is a lack of research investigating the relationship between motor development in adolescence and future, adult performance. This longitudinal study analyzed the three-year development of highly talented young soccer players’ speed abilities and technical skills and examined the relevance of this development to their adult success. The current research sample consisted of *N* = 1,134 players born between 1993 and 1995 who were selected for the German Soccer Association’s TID program and participated in nationwide motor diagnostics (sprinting, agility, dribbling, ball control, shooting) four times between the Under 12 (U12) and Under 15 (U15) age class. Relative age (RA) was assessed for all players, and a total motor score was calculated based on performances in the individual tests. In order to investigate players’ future success, participants were divided into two groups according to their adult performance level (APL) in the 2014/2015 season: Elite (1st-5th German division; *N* = 145, 12.8%) and non-elite players (lower divisions; *N* = 989, 87.2%). Using multilevel regression analyses each motor performance was predicted by Time, Time^2^ (level-1 predictors), APL, and RA (level-2 covariates) with simultaneous consideration for interaction effects between the respective variables. Time and Time^2^ were significant predictors for each test performance. A predictive value for RA was confirmed for sprinting and the total motor score. A significant relationship between APL and the motor score as well as between APL and agility, dribbling, ball control, and shooting emerged. Interaction effects distinctly failed to reach significance. The study found a non-linear improvement in players’ performance for all considered motor performance factors over a three-year period from early to middle adolescence. While their predictive value for future success was confirmed by a significant relationship between APL and most of the considered factors, there was no significant interaction between APL and Time. These findings indicate that future elite players had already been better at the beginning of the TID program and maintained this high level throughout their promotion from U12 to U15.

## Introduction

In order to provide talented young soccer players with an adequate training environment various clubs and associations have implemented comprehensive talent identification and development (TID) programs (e.g., [1, 2]). However, only a small number of young athletes within these programs reach a professional playing level in adulthood [3, 4]. Therefore, a goal-oriented promotion for youth soccer players (i.e., with clearly intended performance outcomes) represents a meaningful challenge. Talent development constitutes a complex process because many different characteristics have an effect on the development of young promising athletes [5]. This complexity stresses the importance of identifying factors that can help predict athletes’ chances of future success, thus providing valuable information for coaches [6, 7].

In the fundamental work of Williams and Reilly [8] (see also [9]), such potential personal talent indicators were categorized into physical, physiological, and psychological factors. Based on this framework, researchers have examined to what extent the various talent factors (isolated as well as in combination) possess predictive power with respect to players’ future success. In various prospective studies, investigators have predominantly assessed the prognostic relevance of motor performance factors, such as sprint, agility, and dribbling performance [10], and found a significant *relationship between motor test performances and future success* in youth soccer (e.g., [11–14]). From a practical perspective, soccer associations also highlight such *speed abilities* (e.g., the ability to move quickly, accelerate or deaccelerate, and change direction; see [15]) *and technical skills* (i.e., on-the-ball performances, including ball control, dribbling, and shooting; see [16]) as particularly relevant [1].

Most of the research in TID has been conducted testing diagnostics’ prognostic validity by *assessing performance only once*. However, talent is a dynamic construct which indicates that developmental aspects must also be taken into account (e.g., [17, 18]). For example, differences in maturation among young players as well as changes in physical or physiological dispositions (e.g., body size or power) may influence the motor parameters’ development and subsequent performance [5, 19, 20]. In addition, Höner et al. [21] reported satisfying inter-individual stabilities of differences for motor diagnostics, even for longer periods of up to three years. Consequently, differential stability could play an important role when investigating the usefulness of assessing motor performance factors in early adolescence [19, 21], which not only represents a period characterized by the beginning of puberty [22], but also the best motor learning phase [23]. Motor performance factors can be subject to particularly meaningful changes within this developmental phase. Due to these potential fluctuations in motor development (e.g., [24]), multiple assessments of the same individuals are needed to describe intra-individual changes and more accurately identify inter-individual differences between young players [25]. Accordingly, various researchers have argued for the use of longitudinal designs, as “monitoring the […] development of talented soccer players over a prolonged period of time can also contribute to an improved understanding and further enhancement of talent development and selection processes” (see p. 585 in [26]). Despite the advantages of longitudinal research, studies using this methodology are rare in talent development research (e.g., [14]).

While a few studies exist in which researchers included multiple measurement points and, simultaneously, combined their assessment with a talent prognosis [25–27], these endeavors are characterized by meaningful limitations. For instance, using a person-oriented approach, Zuber and colleagues [27] investigated 12 to 14-year-old talented soccer players’ development of, among others, speed abilities and technical skills. They found that during a short-term prognostic period of one year highly skilled players reached an elite playing level (i.e., youth national team) significantly more often than their non-highly skilled counterparts. Players were accompanied over a two-year period and their performances at the last assessment (i.e., at the age of 14) were used to investigate differences between participants’ success at the age of 15. However, performance development as a potential career predictor in itself was not addressed. Similarly, utilizing a short-term prognostic period of less than one year after the last assessment, Saward et al. [27] reported significant differences with respect to match-running performance between players who were retained by or released from a youth academy. Although match-running performance was analyzed across three seasons, this study did not address the relationship between performance development over time and the subsequent playing status, which would have required the modelling of interaction effects between these two predictors. The latter was considered in a prospective study by Huijgen and colleagues [26], who found no significant interaction between the dribbling performance development of 14 to 18-year-old athletes and their adult playing level well over two years after the last measurement. Unfortunately, other motor performance factors were not addressed in this study and the average number of measurement points per player (*M* = 1.82, 238 measurements in a total of 131 players) was rather low. Due to these limitations in the current literature, further research with respect to the association between motor development in adolescence and future performance success appears warranted, especially for longer prognostic periods.

Yet, it should also be noted that such research entails *methodological challenges*. First, there are potential issues with the feasibility of these types of studies (e.g., participant dropout) as they, for example, require large sample sizes assessed over a prolonged period of time. Second, conducting research with long-term prognostic periods requires distinct statistical procedures. More specifically, in order to adequately analyze longitudinal data statistical tools are needed that model changes and differences both within and between young players. To assess such longitudinal changes in performance characteristics, Brink et al. [28] suggested using *repeated measures multilevel statistical techniques*. Multilevel modelling provides “more accurate and comprehensive description of relationships in clustered data than do conventional models, by correcting underestimated standard errors, by estimating components of variance at several levels, and by estimating cluster-specific intercepts and slopes” (see p. 1 in [29]). In addition, the application of such models offers the opportunity to consider both group- and individual-level variation in performance development. Unfortunately, to date, only a limited number of researchers have applied these multilevel analyses with longitudinal data in talent development research (e.g., [30]), and even fewer combined them with talent prognosis (e.g., [25, 26]). However, as previously discussed, these studies are also characterized by other limitations. To fill this gap in the literature on TID, the present study was conducted with a longitudinal as well as prospective design within a nationwide TID program.

TID programs usually start their promotion efforts in early adolescence. The German Soccer Association (DFB) selects the top 4% of youth soccer players for its TID program every year beginning with the age class U12 [31]. Subsequently, these highly talented individuals are developed in early adolescence (U12-U15) either in one of the more than 50 youth academies of professional soccer clubs or in one of the DFB’s 366 competence centers. In these competence centers, athletes receive weekly training by a qualified soccer coach which is provided in addition to their regular club training. Focusing on athletes who are promoted at one of the DFB’s competence centers from U12 to U15, the current endeavor was designed to investigate the following two research questions: (1) *How do the speed abilities and technical skills of young elite soccer players develop over the course of individuals’ involvement in the German TID program (U12-U15)*, and (2) *over a long-term prognostic period*, *what predictive power speed abilities and technical skills as well as their development possess with regard to reaching a professional performance level in adulthood*? Thus, the purpose of the present study was to adequately analyze young soccer players’ motor development in early adolescence and its relationship to adult success by utilizing a longitudinal design within a nationwide TID program.

## Methods

### Sample and design

This *three-year longitudinal and eight-year prospective study* analyzed the data of *N* = 1134 male players (birth cohorts 1993, 1994, 1995) who participated in the German TID program from U12 to U15 at one of the DFB’s competence centers between 2004 and 2009. Every year in the fall, each player participated in motor diagnostics, which resulted in four measurement points (i.e., first assessment in U12, following assessments in U13, U14, and U15) for each athlete (4536 total data points from 1134 athletes). Due to the nationwide assessment at the 366 competence centers, diagnostics were conducted within a time window of about seven to eight weeks. The measurement point within this time window was independent of other parameters of the current research, such as age class or performance level. In order to enhance the objectivity and reliability of the assessments across all sites all staff members conducting the tests were provided with a detailed test manual. Additionally, individual sites received practical support through trained research assistants if needed. Nevertheless, the high number of different staff members needed for the nationwide assessments may have contributed to a decrease in reliability. In order to focus on those players who progressed through the full promotion program at the competence centers during adolescence, only individuals who completed the diagnostics’ individual motor test at each of the four measurement points (i.e., U12-U15) were included in this study. Participants were 11.42 ± 0.28 years old at the time of the first measurement point in U12.

Before entering the TID program, players’ parents provided written informed consent for the recording and scientific use of the data collected in the motor diagnostics. In addition, players provided verbal assent before participating in the motor diagnostics. DFB staff members (i.e., coaches with at least the UEFA B-License) conducted the motor tests and, therefore, measured the predictor variables of the present study. The DFB provided the authors with the data for players from the three birth cohorts. The ethics department of the Faculty of Economics and Social Sciences at the first author’s institution and the scientific board of the DFB approved the implementation of this study.

Based on an examination of the rosters of the five highest German leagues [32, 33], the assessed players were divided into two groups according to their *adult performance level (APL*) in the 2014/2015 season. At this time, the investigated athletes were between 19 and 22 years old. This resulted in a long-term prognostic period of six, seven, or eight years, respectively (depending on the birth cohort). Players who appeared in at least one match in one of the five highest German soccer divisions (Bundesliga, 2. Bundesliga, 3. Liga, Regionalliga, Oberliga) constitute the first group. Currently, almost 9,800 athletes are under contract by a club that is part of one of these divisions [34]. Thus, with approximately 3.5 million active soccer players in Germany [35], individuals who compete in one of the upper five German soccer divisions belong to the top 1% of all soccer players in Germany. Therefore, athletes in this group were categorized as *elite* players (*N* = 145, 12.8%). *Non-elite* players (*N* = 989, 87.2%) did not appear in one of the five highest German soccer divisions. Divisions of other countries were not analyzed.

### Measures

The motor test battery consisted of five individual tests. The players were assessed in *sprint* (time for a 20m linear sprint), *agility* (time in a slalom course without a ball) and *dribbling* (time in a slalom course with a ball), *ball control* (time needed to play six passes alternately against two opposing impact walls with at least two ball contacts), and *shooting*. Times for sprint, agility, and dribbling were measured utilizing light barrier systems, whereas times for ball control were assessed with chronographs. Two attempts were conducted for each of these tests, but only the better one was counted. Players were provided with ample time to recover between the attempts. Whereas all these tests are measured by execution time, the shooting test comprises eight shots at two different target fields within the goal (left and right). The shots are rated by a coach with regard to precision (shot on target field represents a hit) and speed (ranked on a three-stage scale). All five individual tests were negatively coded (i.e., a lower value indicated a better performance). Based on the individual test results, a positively coded motor score (i.e., more points indicate better overall performance) was calculated using the following formula (for a detailed description see [21]):

$$\text{Score}=10,000\;*\;{\left[\left(17.29\;*\;\text{sprint}\right)+\left(9.43\;*\;\text{agility}\right)+\left(4.11\;*\;\text{dribbling}\right)+\left(2.41\;*\;\text{ball}\;\text{control}\right)+\text{shooting}\right]}^{-1}$$ (1)

Additionally, each player’s *relative age* (*RA;* measured by the day of birth within a year, i.e., the number of days after January 1^st^) was registered.

Höner and colleagues [21] provided a detailed description of the individual tests and analyzed the test battery’s psychometric properties for a sample comprising nearly 70,000 competence center players (U12-U15). They found excellent internal consistencies in terms of Cronbach’s Alpha (α = .89) as well as satisfying test-retest reliabilities (*r* = .74) for the composite motor score. With regard to the individual tests, they discovered extraordinarily good internal consistencies for sprint (α = .95) and agility (α = .91), whereas these values for ball control (α = .68), dribbling (α = .61), and especially shooting (α = .41) were lower. With the exception of shooting (*r* = .30), test-retest reliabilities ranged between *r* = .50 for ball control and *r* = .76 for sprint.

### Statistical analysis

Data were analyzed using SPSS (Version 24) and R (Version 3.2.2). Pre-analyses of Random-Intercept-Only models revealed an *Intra-Class-Correlation* (*ICC)* of at least 0.10 for the total motor score (*ICC*_*Score*_ = 0.14) and for each single test (*ICC*_*Sprint*_ = 0.27, *ICC*_*Agility*_ = 0.32, *ICC*_*Dribbling*_ = 0.23, *ICC*_*BallControl*_ = 0.10, *ICC*_*Shooting*_ = 0.14). Consequently, in accordance with the recommendations of Kreft and De Leeuw [36] and Teachman and Crowder [37], the longitudinal development of players’ performances in the motor diagnostics was analyzed using multilevel regression analyses.

Two-level regression analyses (Random-Intercept-and-Random-Slope models) were performed for the overall score and each single test. To meet the requirements of the longitudinal data set’s hierarchical structure (i.e., different measurement points nested within different individuals), the repeated measurements were analyzed within (level 1) and between individuals (level 2). In order to describe the developmental changes in each motor performance parameter (MP_ij_) for the player *j* at the measurement point *i* a Random-Intercept-and-Random-Slope model was built:

$${MP}_{ij}\;=\gamma_{00}+\gamma_{10}\;*\;Time+\gamma_{20}\;*\;{Time}^{2}+u_{0j}+u_{1j}\;*\;Time+u_{2j}\;*\;{Time}^{2}+\varepsilon_{ij}$$ (2)

This model included fixed and random effects to account for both players’ overall motor performance development (regarding mean values; i.e., fixed effects) and the unexplained (inter-individual) variation around these means for the intercept and slopes (i.e., random effects; see [38]). More specifically, γ_00_ (intercept, average performance of all individuals at T0), γ_10_ (fixed time slope, mean change in motor performance per year), and γ_20_ (fixed time^2^ slope, mean change in motor performance per year^2^) contributed to the fixed part of the regression formula. The time^2^ slope was added to the model to investigate whether non-linear changes occur in motor performance development. The parameters u_0j_ (random intercept, individual deviation from mean performance γ_00_ at T0), u_1j_ (random time slope, individual deviation from mean time slope γ_10_ per year) and u_2j_ (random time^2^ slope, individual deviation from mean time^2^ slope γ_20_ per year^2^) comprised the random part.

In addition to the time variables (in years after the first assessment in U12, level I predictors), RA (in number of days after January 1^st^) was inserted in the model as level 2 covariate to check whether this variable had an influence on the respective motor performance parameter. Similarly, although APL semantically represents an outcome and not a predictor, this variable was included as level 2 covariate in the model to analyze the differences in developmental changes between the two groups (elite vs. non-elite). Interactions between the time variables and these covariates (e.g., Time x APL) were also investigated to test for any potential relationship between developmental change over time and players’ RA or APL. For each regression analysis (i.e., for each motor performance parameter) all given variables (i.e., random and fixed parts for intercept, Time and Time^2^ as well as RA, APL, and interaction effects) were added to the model and systematically excluded based on model fit changes indicated by -2 log likelihood (deviance). Only significant predictors (α = .05) of the respective motor performance variable were included in the final regression model. Additionally, deviances from the respective final and null model were used to compute Maddala’s *R*^*2*^ to give insight into the explained variance of the regression models [39, 40].

Finally, to also control for cohort effects that may confound the analysis of motor performances’ differences between selection levels [41], two-level regression analyses were performed for each birth cohort separately. These models revealed the same structure of significant predictors for motor performance development as the models based on the data of all three birth cohorts (1993, 1994, and 1995) combined. Therefore, the birth cohorts were accumulated in order to achieve a sufficient number of elite players within the sample and, thus, provide robust results for the developmental changes of the considered motor variables. Only the results for the accumulated data are displayed in the following section.

## Results

*Descriptive statistics* for individuals’ test performances based on APL are presented in Table 1. Generally, players improved in all motor performance parameters within the three-year period from U12 to U15. Furthermore, on average, future elite players performed better than their non-elite counterparts across all measurement points.

**Table 1:** Descriptive statistics of motor performances at the assessed measurement points (U12, U13, U14, U15) for elite (N = 145) and non-elite players (N = 989).

Motor Performance Variable	Group in Adulthood	*M* ± *SD*			
		U12	U13	U14	U15
Score (points)	Non-elite Players	42.37 ± 1.83	44.10 ± 1.92	45.57 ± 1.94	46.68 ± 1.98
	Elite Players	43.08 ± 2.05	45.05 ± 2.10	46.26 ± 2.21	47.48 ± 2.23
	Total Sample	42.46 ± 1.87	44.22 ± 1.97	45.65 ± 1.99	46.78 ± 2.03
20m Sprint (s)	Non-elite Players	3.66 ± 0.17	3.57 ± 0.16	3.46 ± 0.16	3.35 ± 0.17
	Elite Players	3.64 ± 0.18	3.56 ± 0.17	3.44 ± 0.17	3.32 ± 0.17
	Total Sample	3.65 ± 0.17	3.57 ± 0.16	3.46 ± 0.17	3.35 ± 0.17
Agility (s)	Non-elite Players	8.44 ± 0.44	8.22 ± 0.41	8.09 ± 0.37	8.01 ± 0.36
	Elite Players	8.35 ± 0.40	8.10 ± 0.40	8.04 ± 0.38	7.95 ± 0.41
	Total Sample	8.43 ± 0.43	8.21 ± 0.41	8.08 ± 0.37	8.00 ± 0.37
Dribbling (s)	Non-elite Players	11.56 ± 0.83	11.07 ± 0.81	10.74 ± 0.78	10.53 ± 0.73
	Elite Players	11.29 ± 0.72	10.79 ± 0.69	10.55 ± 0.75	10.30 ± 0.65
	Total Sample	11.53 ± 0.82	11.04 ± 0.80	10.72 ± 0.78	10.50 ± 0.72
Ball Control (s)	Non-elite Players	11.73 ± 1.53	10.65 ± 1.47	9.93 ± 1.26	9.48 ± 1.26
	Elite Players	11.34 ± 1.84	10.21 ± 1.34	9.65 ± 1.21	9.25 ± 1.35
	Total Sample	11.68 ± 1.58	10.59 ± 1.46	9.90 ± 1.26	9.45 ± 1.27
Shooting (points)	Non-elite Players	17.83 ± 3.66	16.79 ± 3.74	15.69 ± 3.94	15.04 ± 4.12
	Elite Players	17.31 ± 4.06	15.63 ± 4.19	14.70 ± 4.38	14.09 ± 4.14
	Total Sample	17.76 ± 3.71	16.64 ± 3.82	15.57 ± 4.01	14.92 ± 4.13

Table 2 displays the significant predictors’ regression coefficients of the *multilevel regression analyses*. The explained variances for the individual tests ranged from only 8.8% for shooting to 50.2% for sprinting. An even higher value (53.8%) of explained variance proportion was reached for the score’s regression model. With regard to the random parts of the regression models, best model fits were found when the intercept as well as the time and time^2^ slopes were allowed to vary randomly (except for Time^2^ in sprint and shooting). This indicated substantial inter-individual variation in terms of motor performance at T0 as well as individual variation concerning developmental changes over time.

**Table 2:** Final models’ regression coefficients for each motor performance parameter—Multilevel regression analyses (N = 1134).

Independent Variable		Motor Performance Parameter (Dependent Variable)											
		Score		20m Sprint		Agility		Dribbling		Ball Control		Shooting	
		Coeff.	SE	Coeff.	SE	Coeff.	SE	Coeff.	SE	Coeff.	SE	Coeff.	SE
Fixed effects	Intercept	42.5549***	0.0883	3.6200***	0.0080	8.4340***	0.0132	11.5535***	0.0248	11.7128***	0.0471	17.8963***	0.1129
	Time	1.9118***	0.0634	-0.0896***	0.0048	-0.2436***	0.0143	-0.5436***	0.0293	-1.2193***	0.0579	-1.3182***	0.1660
	Time^2^	-0.1597***	0.0203	-0.0046**	0.0015	-0.0342***	0.0043	0.0682***	0.0091	0.1608***	0.0177	0.1198*	0.0530
	APL	0.7970***	0.1322	ns	-	-0.0728**	0.0262	-0.2423***	0.0497	-0.3181***	0.0808	-0.8936***	0.2109
	RA	-0.0013**	0.0001	0.0002***	0.0000	ns	-	ns	-	ns	-	ns	-
Random effects (SD)	Intercept	1.3238***		0.1396***		0.3406***		0.5569***		1.0865***		1.2431***	
	Time	0.5213***		0.0412***		0.2391***		0.2893*		0.8296***		0.2243*	
	Time^2^	0.1775***		ns		0.0578***		0.0664*		0.1914***		ns	
	Residual	1.3221		0.0998		0.2680		0.6012		1.1272		3.5660	
Explained Variance (in %)		53.8		50.2		23.5		27.5		32.6		8.8	

Note.

*p < .05,

**p < .01,

***p < .001;

ns = not significant (and, therefore, not included in the final model); Coeff. = Estimated Regression Coefficient, SE = Estimated Standard Error, APL = Adult Performance Level, RA = Relative Age.

With respect to the fixed parts of the regression models, the intercept as well as Time and Time^2^ significantly contributed to the prediction of players’ performance in the motor parameters (each *p* < .05). Consequently, individuals’ improvement in the considered talent factors was non-linear within the investigated three-year period. The inserted covariates APL and RA only partly emerged to be significant predictors. Whereas APL was found to be significant for every motor performance parameter (each *p* < .01) with the exception of sprint, RA only reached significance for sprint and the overall score. Furthermore, all considered interaction effects failed to reach significance. Thus, this was also the case for the interaction terms Time x APL as well as Time^2^ x APL, indicating no significant relationship between motor performance development and future success.

**Fig 1:**
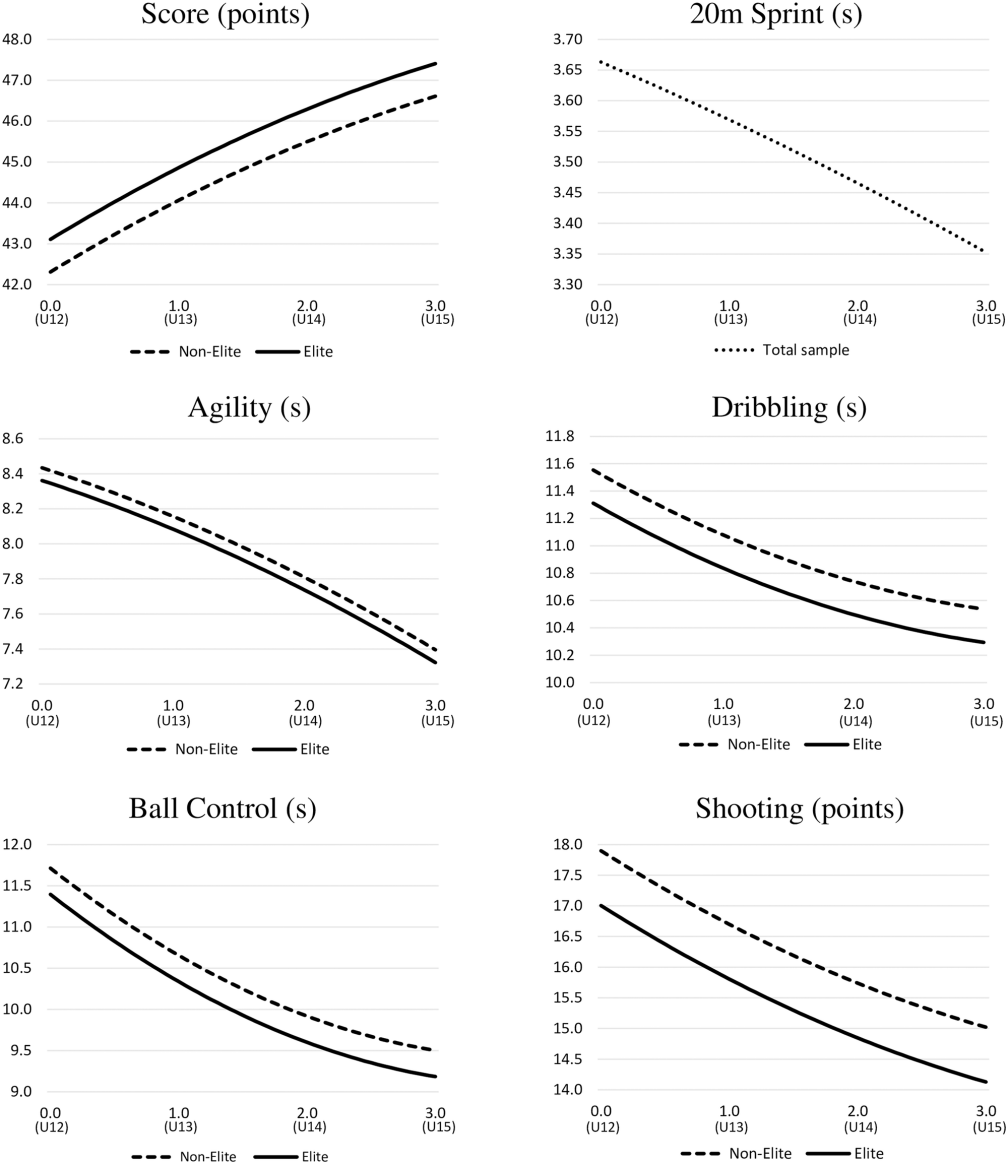
Players’ motor performance development from U12 to U15 predicted by the multilevel regression analyses and separated by adult performance level. Note. Test performances in sprint, agililty, dribbling, ball control and shooting are all negatively coded, that is, a lower value represents a better performance. The x-axis represents the time (in years) from the first measurment point in U12 (Time = 0) to the last assessment in U15 (Time = 3). Individuals’ development for the sprint test is displayed independently of APL, because this variable was not found to be a significant predictor for participants’ sprint performance.

In order to gain insight into youth players’ motor development during their time in the TID program, their motor performance can be calculated (based on time after the first measurement point, RA, and/or APL) using the results for the fixed parts from the regression analyses (see Table 2). The estimated curves for the respective talent factors (see Fig 1) show players’ average motor development within the investigated three-year period from U12 (Time = 0) to U15 (Time = 3). For example, the following best model (derived from the Eq (2) described in the statistical analysis section) can describe the overall motor performance development represented by the score:

$$\text{Score}\;\text{performance}=42.55+1.91\;*\;\text{Time}–0.16\;*\;{\text{Time}}^{2}+0.80\;*\;\text{APL}–0.001\;*\;\text{RA}$$ (3)

Regarding the independent variables within this formula, Time represents the period of time after the first measurement point in U12 (in years). For instance, for the third assessment in U14, Time is equal to “2”. APL is set to “1” if elite players’ dribbling performance is predicted and to “0” for non-elite players. RA ranges from 1 (player born January 1^st^) to 365 (players born December 31^st^). For instance, a player’s score performance improves (on average) by 1.75 points (= 1.91–0.16) within the first year of the promotion (1.87 ≤ *SD*_*Score*_ ≤ 2.03 for the four age classes U12 –U15) and by 3.18 (= 1.91 * 2–0.16 * 2^2^) points within the first two years. Across all investigated measurement points future elite players performed 0.80 score points better than their non-elite counterparts. Lastly, with respect to RA, an athlete born January 1^st^ has a score performance that is 0.364 (= 364 * 0.001) points better than a player born December 31^st^.

## Discussion

Recent research offers a broad understanding of the *usefulness of motor talent diagnostics to predict future success* (e.g., [10]). More specifically, researchers have provided information about the prognostic validity of motor performance factors for future performance level (e.g., [4, 11]). Due to the fact that talent is considered to be a dynamic construct [17] and because previous studies have generally been conducted assessing parameters only once, this investigation’s focus was the development of motor performance factors within a nationwide TID program as well as its predictive value for adult success. By utilizing a three-year longitudinal design that included four measurement points for a large sample of athletes (i.e., providing sufficient test power), the present endeavor provides more reliable insight into young soccer players’ motor performance development in early adolescence. Additionally, by applying multilevel regression techniques for analyzing longitudinal data, this study used statistical procedures that allowed for a consideration of inter-individual differences (such as APL or RA) and provided information about intra-individual change. Thus, variation in motor development between players of different future performance levels over time (level 2) as well as athletes’ individual development (level 1) over a three-year period were comprehensively analyzed.

### Motor performance factors’ prognostic validity

The current study confirmed the prognostic validity of the presented motor test battery for adult success (6–8 years later). A significant difference was found between future elite and non-elite players’ overall motor score assessed within early to middle adolescence as well their respective speed abilities (agility) and technical skills (dribbling, ball control, and shooting). These results support the current state of research concerning the predictive power of motor performance (e.g., [4, 12, 13, 42]) and can likely be explained by the essential impact speed abilities and technical skills have on match-winning situations in soccer [9, 13]. For instance, a better agility or dribbling performance (indicated by a better time in the diagnostics) may result in a more ideal position on the pitch for winning or defending the ball against an opponent. This advantage may exist with regard to players’ current performance within early adolescence, and also manifest itself in their likelihood to be further promoted in middle to late adolescence. Furthermore, due to the sufficient long-term stabilities for the motor score investigated in this study (r_U12/U15_ ≈ .51; [43]), a better performance in early adolescence may also be linked to an increased probability of superior performance in adulthood. However, despite the prognostic relevance of motor performance, there was no statistically significant relationship of motor performance development and participants’ future playing level in adulthood. These findings are consistent with the conclusions of [26], suggesting that players who reach an elite level in adulthood, on average, already possess advanced motor abilities upon entry into the TID program. In other words, they were able to maintain their advantage over future non-elite players throughout the stages of promotion.

### Motor development

Nevertheless, a substantial improvement from U12 to U15 was detected in participants’ performance across all investigated motor performance factors. This progression was *non-linear*, as players’ motor performance increased considerably more in the first year of their promotion (i.e., U12 to U13) than in subsequent years (i.e., U13 to U14 or U14 to U15). These findings are mostly in line with previous research (e.g., [42, 44]). Most recently, Fransen and colleagues [45] found that participants’ motor competence developed faster before they reached the age of peak height velocity. Similarly, Huijgen et al. [44] indicated a meaningful improvement of players’ dribbling performance between the ages of 12 and 14, followed by a phase where this development plateaued. It is likely that this non-linear development is due to the rapid biological, maturational changes in individuals during early adolescence. An alteration in body composition (e.g., changing hormones, metabolic rhythms) can be accompanied by shifts in motor dispositions. In fact, athletes’ physical or physiological dispositions are expected to suddenly change when they go through puberty during early adolescence [5, 46].

The effect of RA, in general, was found to be rather low for most of the motor performance factors in the present study. Except for sprinting and the total motor score, this variable did not show a significant influence on players’ performance. Nevertheless, in contrast to Votteler and Höner’s suggestion [47], findings of the present study could not confirm an effect of RA on motor performance development. Therefore, additional focus should be given to the influence of relative age characteristics in future research. Although RA seemed to play a limited role in the motor development of elite and non-elite athletes (which is in line with Höner et al. [4]), it seems likely that some future elite players were already advanced in maturation (independent of their relative age). This advantage, among other implications, may have resulted in possessing better speed abilities and technical skills compared to those individuals who mature later [48]. However, these maturational aspects were not part of the present study and, therefore, *other age-related considerations* such as biological maturity (e.g., skeletal age or age at peak high velocity; see [49]), which may provide additional insight into inter-individual developmental differences, should be explored in future research.

Further attention should also be given to the *individual differences* that was found with respect to athletes’ motor performance development over time. The random effects in multilevel modelling indicated significant *inter-individual variations* between players with regard to their baseline performance at the beginning of the TID program (i.e., random intercept) as well as with regard to their motor performance development over time (i.e., random slope). This finding is particularly meaningful given the preselected, homogenous subsample of athletes who belong to the top 4% of youth soccer players in Germany. Similar inter-individual differences in young soccer players’ motor development were found in longitudinal studies about dribbling [26] and match-running performance [25].

Furthermore, while no interaction between motor performance development and a player’s APL materialized, these results were derived from a group-based perspective (i.e., based on the average player within the investigated sample). The detected variation between players as well as the different measurement points potentially allows for such a relationship to emerge on an individual level. Thus, it seems likely that single case analyses of developmental pathways—in addition to group based considerations—will provide a more in-depth understanding of youth soccer players’ motor development in early adolescence. For instance, over 20% of the investigated elite players in the current study had a lower motor score performance in U12 than the average non-elite player. The consideration of such specific types of players (e.g., athletes who reach an elite adult performance level although they demonstrated a below-average performance at the outset of promotion) might be useful to gain more knowledge about the individual development of talented young soccer players.

### Limitations

The current study demonstrated the general prognostic relevance of motor performance factors for adult success. Yet there were no significant interactions between the non-linear motor development and future performance level. Additionally, RA generally showed a rather small impact on motor performance development. Despite the usefulness of these findings, there are various limitations that should be addressed in future research.

First, as previously described, *selection effects* (i.e., the sample consisted of individuals who belong to the top 4% of youth soccer players of the U12 age class in Germany) could have affected the current sample’s constitution regarding its homogeneity [50]. In addition, only players who performed the motor tests regularly (once a year) during their three-year promotion from U12 to U15 were included in the study. Accordingly, players who transferred to a youth academy within the investigated time span or dropped out of the TID program were not considered. It seems likely that players who reached the youth academy level belonged to the better motor performers (e.g., see [21]), whereas players who dropped out were presumably athletes with lower motor performances. This possibly resulted in a more homogenous subgroup of players with similar motor development within the promotion program at the DFB’s competence centers. This study focused on the development of competence center players who were part of the complete promotion program (U12-U15). Based on this investigation, future research should address issues of homogeneity by, for instance, analyzing the motor development of players who dropped out to gain insight into potential similarities and differences with those individuals who stayed in the TID program.

Second, while the current study longitudinally assessed young athletes’ motor performance, it should be noted that *environmental factors* might play an important role during an athlete’s development in early adolescence. For instance, coaches have a meaningful influence on physical and psychological development [51]. With respect to the current findings, coaches likely differ in the training programs they provide or the contextual environment they nurture. Thus, such social facets should be included in future research addressing inter-individual variation with respect to motor development.

Third, the developmental periods in which talent factors are assessed can have a meaningful impact on their predictive value [52]. Thus, the different motor dimensions that have been addressed within this study (speed and technique) might also show a specific (e.g., quick, deaccelerated, or abrupt) development in earlier and later age classes which may, in turn, affect their predictive value (e.g., [4, 6]). For instance, as mentioned before, Philippaerts et al. [53] stated that phases of accelerated and deaccelerated development could occur just before and after the age of peak height velocity (see also [45]) and might influence talent factors’ discriminating power. The current results are based on data from a sample of 11 to 14-year-old individuals, and, therefore, similar investigations should be conducted with youth players from other *developmental phases in adolescence*.

Last, the current study only focused on two dimensions of talent factors (i.e., speed abilities and technical skills) and analyzed their influence on individuals’ future success. Development of these motor performance factors was found to be quite similar for elite and non-elite players. It is possible that the investigated non-elite players spend additional time and effort on improving their technical skills and speed abilities during their promotion in the TID program in order to continue to be competitive with their elite counterparts. However, Huijgen et al. [26] argued that due to this additional focus on improving their motor performance, these players’ may not be able to sufficiently develop other skills that would be necessary to reach the top level in adulthood. Given this concern, it seems necessary to “leave the one-dimensional arena and focus on a multidimensional playing field” (see p. 7 in [18]) by conducting research that investigates a more complex and multidimensional array of variables. For example, recent studies illustrated the predictive power of various potential relevant talent facets, including psychological attributes such as motivational and volitional components (e.g., [54]) or tactical competences such as positioning and deciding (e.g., [55]). By pursuing a multidimensional pathway, it may be possible to investigate whether specific weaknesses in one of the different attributes may be compensated by exceptional strengths in others [18].

## Conclusion

This study provides reliable empirical evidence of the prognostic relevance of speed-related and technical skill tests within a nationwide TID program. The results demonstrated prognostic validity of motor performance factors over a long-term period (≈ eight years). However, the three-year development of these characteristics did not correspond significantly with the adult performance level, indicating that future successful players already possessed advanced motor abilities upon entry into the TID program and were able to maintain their advantage over future non-elite players throughout the program. Nevertheless, the limitations discussed in this paper coupled with the fact that there have been few studies on the prognostic relevance of performance development in soccer highlight a significant need for further research.

## Supporting information

S1 File Data set. (TXT)
